# Predição de Obstrução Coronariana Significativa em População com Suspeita de Doença Coronariana e Ausência de Cálcio Coronariano: CORE-64 e CORE320

**DOI:** 10.36660/abc.20220183

**Published:** 2023-03-07

**Authors:** Anderson C. Armstrong, Rodrigo Cerci, Matthew B. Matheson, Tiago Magalhães, Satoru Kishi, Jeff Brinker, Melvin E. Clouse, Carlos E. Rochitte, Christopher Cox, João A. C. Lima, Armin Arbab-Zadeh

**Affiliations:** 1 Universidade Federal do Vale do São Francisco Petrolina PE Brasil Universidade Federal do Vale do São Francisco , Petrolina , PE – Brasil; 2 Johns Hopkins Hospital Baltimore EUA Johns Hopkins Hospital , Baltimore – EUA; 3 Johns Hopkins Bloomberg School of Public Health Baltimore EUA Johns Hopkins Bloomberg School of Public Health , Baltimore – EUA; 4 Beth Israel Deaconess Medical Center Boston MA EUA Beth Israel Deaconess Medical Center , Boston , MA – EUA; 5 Instituto do Coração Hospital das Clínicas Universidade de São Paulo São Paulo SP Brasil Instituto do Coração do Hospital das Clínicas da Universidade de São Paulo , São Paulo , SP – Brasil

**Keywords:** Doença Arterial Coronariana/complicações, Escore de Cálcio, Tomografia Computadorizada por Raios X/métodos, Angiografia Coronária, Diagnóstico por Imagem/métodos, Dor Torácica

## Abstract

**Fundamento:**

A avaliação do Escore de Cálcio Coronariano (ECC) pode ser realizada por tomografia computadorizada sem contraste para prever eventos cardiovasculares, mas tem menor valor na estratificação de risco em pacientes sintomáticos.

**Objetivo:**

Identificar e validar preditores de obstrução coronariana significativa (OCS) em pacientes sintomáticos sem calcificação da artéria coronária.

**Métodos:**

Um total de 4258 participantes foram rastreados dos estudos CORE64 e CORE 320, nos quais foram avaliados pacientes encaminhados para angiografia invasiva, e do Quanta Registry que incluiu pacientes encaminhados para angiotomografia. Modelos de regressão logística avaliaram associações entre fatores de risco cardiovascular, ECC e OCS. Um nível de significância de 5% foi usado nas análises.

**Resultados:**

Dos 509 participantes do estudo CORE, 117 (23%) apresentaram um ECC igual a zero; 13 (11%) pacientes sem cálcio coronariano apresentaram OCS. A ausência de cálcio coronariano correlacionou-se com idade mais jovem, sexo feminino, índice de massa corporal mais baixo, ausência de diabetes, e ausência de dislipidemia. O fato de ser fumante atual aumentou em 3,5 vezes a probabilidade de OCS e outros fatores de risco cardiovasculares não apresentaram associação significativa. Considerando os achados clínicos, um algoritmo para estratificar os pacientes com ECC igual a zero foi proposto, e tiveram desempenho limitado na coorte de validação (AUC 58; IC95% 43, 72).

**Conclusão:**

Um perfil de risco cardiovascular mais baixo está associado a um ECC igual a zero em pacientes de alto risco. Tabagismo é o preditor mais forte de OCS em pacientes com ausência de cálcio coronariano.

## Introdução

A angiografia coronariana por tomografia computadorizada (ACTC) permite a detecção precisa, não invasiva, de doença arterial coronariana (DAC) ^1 , 2^ e facilita a estratificação de risco em pacientes com dor torácica. ^3^ Contudo, a preocupação com a exposição à radiação e ao contraste ainda permanece. ^4 - 6^

O escore de cálcio coronariano (ECC) pode ser avaliado usando a tomografia computadorizada (TC) sem contraste, com uma exposição muito baixa à radiação. Resultados obtidos de pacientes assintomáticos, de baixo risco, mostram que a ausência de cálcio coronariano está associada a uma baixa probabilidade de obstrução coronariana significativa (OCS) e um desfecho favorável do paciente. ^7^ Porém, esses achados não se aplicam a populações sintomáticas, de alto risco, em que a OCS pode estar presente em mais de 10% dos paciente com ECC igual a zero. ^8 , 9^

A presente investigação foi realizada para identificar preditores válidos de OCS em pacientes sintomáticos sem calcificação da artéria coronária.

## Métodos

### Delineamento e população do estudo

Um total de 4258 indivíduos de três coortes foram rastreados para a inclusão do estudo. Combinamos participantes sem história de DAC incluídos nos estudos CORE 64 e CORE 320, que possuem populações com características quase idênticas, para determinar a prevalência e os preditores de OCS em pacientes sem cálcio nas artérias coronárias. Um registro realizado em um único centro foi usado para validar o modelo dos preditores. Os métodos são apresentados em detalhes nos materiais suplementares.

Em resumo, os estudos “Coronary Artery Evaluation Using 64-Row Multidetector Computed Tomography Angiography (CORE-64)” e o “Coronary Artery Evaluation Using 320-row Multidetector Computed Tomography Angiography and Myocardial Perfusion (CORE320)” são estudos prospectivos que incluíram participantes com suspeita ou diagnóstico confirmado de DAC, encaminhados para angiografia coronária convencional. Os métodos dos estudos CORE-64 e CORE320 foram publicados detalhadamente anteriormente. ^2 , 10^ Finalmente, o Quanta Registry é um registro de pacientes encaminhados para ACTC para fins clínicos em um único centro. Os participantes do Quanta Registry foram incluídos na coorte de validação se relatassem dor torácica.

Todos os estudos foram aprovados pelos comitês de ética da instituição local, e todos os pacientes assinaram um termo de consentimento.

### Aquisição e análise dos dados da TC das artérias coronárias

**Figura 1:**
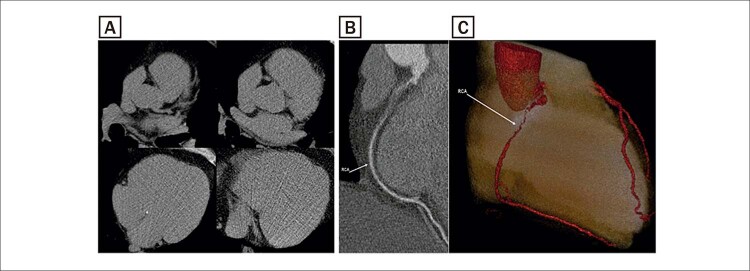
– Escore de cálcio coronariano e angiotomografia de um participante do estudo CORE que apresentava obstrução coronariana significativa escore de cálcio igual a zero. A) Escore de cálcio coronariano. B) Angiotomografia (reconstrução multiplanar). C) Angiotomografia (reconstrução em 3D).

No CORE64, a aquisição de imagens para o ECC foi realizada por um aparelho de TC com 64 canais (Aquilion, Toshiba Medical Systems), ^2^ e no CORE320, por um scanner 320-MDCT (Aquilion ONE, Toshiba Medical Systems). ^11^ Em ambos os casos, as imagens foram analisadas para o ECC em um único centro, por profissionais experientes, cegos quanto a informação clínica do paciente ( Figura 1 ).

No Quanta Registry, as imagens foram adquiridas usando um tomógrafo de 128 canais (iCT sp, Philips Healthcare). As imagens foram revisadas por dois profissionais experientes. Um relatório clínico final foi gerado para cada paciente, que foi classificado, segundo resultado das análises: normal (sem DAC), DAC não obstrutiva (<50% estenose), ou DAC obstrutiva (>50% estenose), o que também foi registrado no basal.

### Aquisição e análise dos dados da angiografia coronária invasiva

Nos estudos CORE, angiografia coronária invasiva (ACI) foi realizada segundo indicação clínica, usando técnicas padrões, dentro de 60 dias da aquisição da TC combinada. A gravidade da lesão foi determinada por angiografia coronária quantitativa (ACQ), como descrito anteriormente. ^2^ OCS foi definida como uma obstrução igual ou maior que 50%, medida por ACQ invasiva.

### Análise estatística

As variáveis contínuas foram apresentadas em mediana e intervalo interquartil. As variáveis qualitativas foram apresentadas em frequências (absoluta e relativa). O programa STATA 10.0 foi usado para as análises. A distribuição dos dados foi avaliada por gráficos, incluindo histogramas e gráficos Q-Q.

Primeiramente, modelos de regressão logística univariada e multivariada avaliaram fatores de risco cardiovasculares (CV) e dados antropométricos como preditores de ausência de cálcio coronariano (variável dependente) na coorte inteira e nos pacientes com OCS. Em seguida, modelos de regressão logística univariada e multivariada avaliaram o subgrupo de pacientes sem cálcio coronariano para avaliar os preditores de OCS (variável dependente). Os resultados dos modelos de regressão logística estão apresentados como odds ratio (e intervalo de confiança de 95%). Por fim, um algoritmo foi gerado para predição de OCS por ACI em pacientes sem cálcio coronariano por TC e então validado em uma coorte diferente por validação cruzada de 10 vezes na amostra original.

O tamanho amostral dos estudos CORE-64, CORE-320, e Quanta Registry foram computados. Incluímos todos os participantes desses estudos com dados disponíveis. Um nível de significância de 5% foi usado nas análises.

## Resultados

### Prevalência e preditores de OCS em pacientes sem cálcio coronariano

Características dos 509 participantes (273 do estudo CORE64 e 236 do estudo CORE320) estão apresentadas na Tabela 1 . A maioria dos participantes incluídos eram homens na sétima década de vida, com uma alta prevalência de fatores de risco CV. Desses 509, 117 (23%) apresentaram um ECC de Agatston de zero. Um total de 252 (49%) pacientes e 13 (11%) dos pacientes sem cálcio coronariano apresentaram pelo menos uma OCS. Por outro lado, 392 (64%) dos pacientes com ECC ≥ 1 apresentaram DAC obstrutiva.

**Tabela 1:** – Características dos pacientes nos estudos CORE64 e CORE320

Preditor	Mediana [IIQ] ou n (%)		

	CORE-64	CORE320	Total

	(n=273)	(n=236)	(n=509)
Idade	60 [53, 67]	62 [57, 68]	61 [55, 67]
Sexo masculino	199 (73%)	136 (58%)	335 (66%)
**Etnia**			
Branca	192 (70%)	138 (58%)	330 (65%)
Afro-americana	13 (5%)	29 (12%)	42 (8%)
Asiática	58 (21%)	66 (28%)	124 (24%)
Outro	10 (4%)	3 (1%)	13 (3%)
IMC, Kg/m ^2^	27 [25, 30]	27 [24, 31]	27 [25, 30]
Hipertensão	193 (71%)	167 (71%)	360 (71%)
Diabetes	62 (23%)	88 (37%)	150 (29%)
Dislipidemia	165 (60%)	138 (60%)	303 (60%)
**Tabagismo**			
Nunca fumou	119 (44%)	107 (47%)	226 (45%)
Ex-fumante	108 (40%)	72 (32%)	180 (36%)
Fumante atual	46 (17%)	47 (21%)	93 (19%)
História familiar de DAC	75 (27%)	96 (42%)	171 (34%)

*IMC: índice de massa corporal; DAC: doença arterial coronariana.*

O subgrupo de pacientes sem calcificação coronariana era, em geral, mais jovem, do sexo feminino, com Índice de Massa Corporal (IMC) mais baixo, sem diabetes, e sem dislipidemia.

Preditores de ECC igual a zero em pacientes com OCS estão apresentados na Tabela Suplementar 1. Nessa população, idade mais jovem e sexo feminino foram consistentemente relacionados com ausência de cálcio coronariano. Fatores de risco CV não apresentaram associação significativa com cálcio coronariano em pacientes com OCS.

### Avaliação de obstrução coronária significativa na ausência de cálcio coronariano

A predição de OCS por antropometria e fatores de risco CV em pacientes sem cálcio coronariano está apresentada na Tabela 2 . Nesses achados, a OCS foi associada tanto com ACTC quanto com ACI. Tabagismo atual aumentou em 3,5 vezes a chance de OCS em pacientes sem cálcio coronariano. Outros fatores de risco CV não foram significativamente associados com OCS nessa população. Com base nesses modelos preditivos para CORE64 e CORE320, foi criado um algoritmo clínico para excluir OCS (material suplementar).

**Tabela 2:** – Predição de obstrução coronariana significativa nos participantes com escore de cálcio coronariano igual a zero, medido por angiografia coronariana por tomografia computadorizada (ACTC) e por angiografia coronária invasiva (ACI)

Preditor	ACTC ≥ 50%; n=134		ACI ≥ 50%; n=134	
	
	Odds Ratio (IC95%)		Odds Ratio (IC95%)	
	
	Univariada	Multivariada	Univariada	Multivariada
Idade, anos	0,956 (0,900; 1,016)	0,960 (0,888; 1,038)	0,979 (0,927; 1,034)	0,987 (0,924; 1,054)
Sexo masculino	1,922 (0,715; 5,165)	1,382 (0,415; 4,596)	1,977 (0,777; 5,032)	1,558 (0,524; 4,632)
**Etnia**				
Afro-americana vs. Branca	0,227 (0,028; 1,839)	0,148 (0,016; 1,342)	0,369 (0,078; 1,741)	0,327 (0,063; 1,684)
Asiática vs. Branca	0,769 (0,230; 2,578)	0,706 (0,162; 3,073)	0,596 (0,182; 1,955)	0,442 (0,108; 1,800)
Outra vs. Branca	3,333 (0,506; 21,970)	4,260 (0,470; 38,601)	0,969 (0,101; 9,276)	1,169 (0,102; 13,336)
IMC, Kg/m ^2^	1,019 (0,922; 1,126)	0,986 (0,852; 1,140)	0,991 (0,900; 1,091)	0,939 (0,819; 1,076)
Hipertensão	1,727 (0,613; 4,860)	1,977 (0,576; 6,787)	1,361 (0,529; 3,503)	1,602 (0,519; 4,942)
Diabetes	1,831 (0,632; 5,302)	2,329 (0,652; 8,319)	1,917 (0,700; 5,245)	2,458 (0,773; 7,823)
Dislipidemia	2,132 (0,793; 5,731)	1,932 (0,614; 6,081)	1,150 (0,469; 2,822)	1,160 (0,410; 3,282)
**Status de tabagismo**				
Fumante atual vs. ex-fumante/nunca fumou	3,630 (1,301; 10,128)	3,562 (1,036; 12,246)	3,630 (1,358; 9,706)	3,448 (1,112; 10,691)
História familiar de DAC	1,677 (0,628; 4,474)	1,862 (0,568; 6,105)	1,293 (0,501; 3,340)	1,347 (0,454; 3,997)
**Overall AUC**		**78 (75-93)**		**73 (70-89)**

*Os tamanhos das amostras refletem a análise multivariada, em que os indivíduos com qualquer covariável faltante são excluídos. Os tamanhos das amostras nas análises univariadas podem ser maiores. IMC: índice de massa corporal; DAC: doença arterial coronariana.*

Na coorte de validação de 742 indivíduos, somente 16 (2,2%) apresentaram doença pela ATC. A maioria (77,2%) apresentou angina atípica, 12,5% apresentaram angina típica, e 10,2% apresentaram dor torácica não anginosa, com um perfil de risco CV em geral mais baixo em comparação aos estudos CORE ( Tabela 3 ). Após aplicar o algoritmo CORE, 199 (26,8%) dos 742 pacientes foram classificados como apresentando doença coronariana significativa. Os resultados da validação estão resumidos na Tabela Suplementar 2.

**Tabela 3:** – Características dos participantes com escore de cálcio escore igual a zero nos estudos CORE e na coorte de validação

Característica	Mediana [IQR] ou n (%)	

	CORE (n=117)	Coorte de validação (n=742)
Idade, anos	56 [51, 62]	52 [43, 61]
Sexo masculino	54 (46%)	246 (33%)
IMC, Kg/m ^2^	27 [24, 30]	27 [24, 30]
Hipertensão	67 (58%)	322 (43%)
Diabetes	22 (19%)	85 (11%)
Dislipidemia	54 (46%)	278 (37%)
Tabagismo atual	21 (18%)	59 (8%)
História familiar de DAC	34 (29%)	181 (24%)

*IMC: índice de massa corporal; DAC: doença arterial coronariana.*

## Discussão

Nosso estudo demonstrou que idade mais jovem, sexo feminino, e perfil de risco CV mais baixo foram associados com ECC igual a zero em pacientes de alto risco encaminhados para cateterismo cardíaco invasivo. Também mostramos que, nessa população, apresentar um ECC igual a zero associou-se a um risco 83% mais baixo de OCS em comparação a qualquer risco de calcificação coronariana. Destaca-se o fato de que tabagismo atual tenha sido o preditor mais forte de OCS em pacientes sem cálcio coronariano.

A doença coronariana é uma causa importante de morte em todo o mundo. Esforços preventivos têm sido feito para o diagnóstico precoce de DAC, bem como para a estratificação precisa do risco de eventos coronários. ^12^ Em pacientes assintomáticos, a presença e o grau de cálcio coronariano mostraram-se um forte preditor de eventos CV; ^13^ no entanto, o papel do cálcio coronariano para a tomada de decisão, particularmente em pacientes sintomáticos, não está bem estabelecido. ^14^

Em concordância com estudos anteriores, ^13^ mostramos que a ausência de cálcio coronariano reduz a probabilidade de OCS. Estudos clínicos recentes, contudo, sugerem que detectar DAC não obstrutiva pode ser tão importante quanto detectar OCS para direcionar o manejo do paciente. ^15 , 16^ Ainda existem controvérsias sobre o papel do cálcio coronariano na exclusão de doença coronária significativa em pacientes sintomáticos. ^17^

Como esperado, observamos que mulheres mais jovens com dor torácica, mas sem diabetes, dislipidemia ou hipertensão apresentaram maior probabilidade de apresentarem um ECC igual a zero. Essa é uma população frequentemente vista em salas de emergências. Grandhi et al., ^18^ encontraram uma prevalência de OCS abaixo de 5% em um registro de pacientes sem cálcio coronariano. Contudo, os médicos podem não estar aptos a dar alta a pacientes sintomáticos com base somente na ausência de cálcio nas artérias coronárias. Assim, está clara a necessidade de desenvolvermos outras avaliações, tal como um escore clínico, para melhor estratificar o risco nesse grupo de pessoas com ECC igual a zero. ^19^

Ao analisar os parâmetros associados com OCS nos indivíduos sem cálcio coronariano, o tabagismo atual destaca-se de maneira consistente como um fator de risco importante. Depósitos de cálcio ocorrem precocemente na cascata da doença aterosclerótica, mas são detectados por exame de imagem somente mais tardiamente. ^20^ É possível que o tabagismo acelere a doença coronariana levando a uma obstrução significativa dos vasos antes que os depósitos de cálcio ocorram. Ainda, o tabagismo correlaciona-se fortemente com inflamação e trombose, as quais podem ser fatores importantes relacionados com a instabilidade de placas ateroscleróticas, e posterior obstrução repentina dos vasos. ^21^

Outros fatores de risco, como idade, parecem ser relevantes na avaliação da capacidade prognóstica do ECC. ^22^ Por isso, nós combinamos tabagismo com idade e fatores de risco tradicionais e propomos um algoritmo que pudesse estratificar o risco de OCS em pacientes com ECC igual a zero. Entretanto, a capacidade discriminatória diminuiu na coorte de validação. A validação perdeu sensibilidade para avaliar o evento relativamente raro de OCS, mas manteve especificidade e valor preditivo negativo favoráveis. Assim, embora o algoritmo não seja viável para uma ampla utilização no momento, um algoritmo clínico pode ser útil para identificar melhor os pacientes sem cálcio coronariano que não são submetidos à ACTC. Um ensaio clínico poderia ajudar a estabelecer a melhor abordagem que incluísse o ECC em pacientes sintomáticos.

Nosso estudo possui limitações importantes. Apesar de uma amostra relativamente grande, a OCS é um evento raro em pacientes sem cálcio coronariano, e o baixo número de desfechos pode comprometer a análise estatística. No entanto, a doença coronária obstrutiva foi relativamente rara em pacientes sem cálcio coronariano, e difícil de se prever usando variáveis clínicas. Existem limitações metodológicas intrínsecas usando QUANTA Registry como uma coorte de validação para um algoritmo de risco desenvolvido nos estudos CORE. O fato de o algoritmo não ter apresentado um desempenho tão bom na coorte de validação como foi observado nos estudos CORE pode ser explicado pela prevalência mais baixa de doença, afetando a sensibilidade e o valor preditivo positivo.

### Aspectos éticos

Todos os centros envolvidos nos estudos CORE-64 e CORE320 receberam aprovação dos comitês éticos institucionais locais, e todos os pacientes assinaram um termo de consentimento. Todos os pacientes do Quanta Registry assinaram um termo de consentimento e autorizaram o uso de seus dados para fins de pesquisa. Ainda, os estudos foram conduzidos de acordo com os padrões éticos estabelecidos na Declaração de Helsinki de 1964 e suas modificações posteriores, ou com padrões éticos comparáveis.

## Conclusões

A ausência de cálcio nas artérias coronárias reduz o risco de OCS. O tabagismo foi um fator de risco importante relacionado à OCS na ausência de cálcio coronariano. Nós propomos um escore de risco que inclui variáveis clínicas, e tem como objetivo excluir doença coronariana em pacientes sintomáticos e ECC igual a zero. No entanto, mais estudos são necessários para comprovar esse conceito.

## *

Material suplementar

Para informação adicional, por favor, clique aqui
