# Supplementary material for: Predição de Obstrução Coronariana Significativa em População com Suspeita de Doença Coronariana e Ausência de Cálcio Coronariano: CORE-64 e CORE320
Source: Arq Bras Cardiol. 2023 Mar 7;120(3):e20220183. [Article in Portuguese] doi: 10.36660/abc.20220183 (PMC12080605; doi:10.36660/abc.20220183)
Supplement: Translation

# Translation: Predicting Significant Coronary Obstruction in a Population with Suspected Coronary Disease and Absence of Coronary Calcium: CORE-64 / CORE320 Studies

**Authors:** Anderson C. Armstrong, Rodrigo Cerci, Matthew B. Matheson, Tiago Magalhães, Satoru Kishi, Jeff Brinker, Melvin E. Clouse, Carlos E. Rochitte, Christopher Cox, João A. C. Lima, Armin Arbab-Zadeh

**Affiliations:** 1 Brasil Universidade Federal do Vale do São Francisco, Petrolina, PE – Brasil; 2 EUA Johns Hopkins Hospital, Baltimore – EUA; 3 EUA Johns Hopkins Bloomberg School of Public Health, Baltimore – EUA; 4 EUA Beth Israel Deaconess Medical Center, Boston, MA – EUA; 5 Brasil Instituto do Coração do Hospital das Clínicas da Universidade de São Paulo, São Paulo, SP – Brasil

**Keywords:** Coronary Artery Disease/complications, Indice Calcio, Tomography, X Ray Computed/methods, Coronary Angiography, Diagnostic Imaging/methods, Chest Pain

## Abstract

**Background:**

Coronary artery calcium (CAC) scanning can be performed using non-contrast computed tomography to predict cardiovascular events, but has less value for risk stratification in symptomatic patients.

**Objective:**

To identify and validate predictors of significant coronary obstruction (SCO) in symptomatic patients without coronary artery calcification.

**Methods:**

A total of 4,258 participants were screened from the CORE64 and CORE320 studies that enrolled patients referred for invasive angiography, and from the Quanta Registry that included patients referred for coronary computed tomography angiography (CTA). Logistic regression models evaluated associations between cardiovascular risk factors, CAC, and SCO. An algorithm to assess the risk of SCO was proposed for patients without CAC. Significance level of 5% was used in the analyses.

**Results:**

Of the 509 participants of the CORE study, 117 (23%) had zero coronary calcium score; 13 (11%) patients without CAC had SCO. Zero calcium score was related to younger age, female gender, lower body mass index, no diabetes, and no dyslipidemia. Being a current smoker increased ~3.5 fold the probability of SCO and other CV risk factors were not significantly associated. Considering the clinical findings, an algorithm to further stratify zero calcium score patients was proposed and had a limited performance in the validation cohort (AUC 58; 95%CI 43, 72).

**Conclusion:**

A lower cardiovascular risk profile is associated with zero calcium score in a setting of high-risk patients. Smoking is the strongest predictor of SCO in patients without CAC.

## Introduction

Coronary computed tomography angiography (CCTA) allows accurate, noninvasive detection of coronary artery disease (CAD)^1 , 2^and facilitates risk stratification in patients presenting with chest pain.^3^Nevertheless, concerns about radiation and contrast exposure remain.^4 - 6^

Coronary artery calcium (CAC) scanning can be performed using non-contrast computed tomography (CT) with very low radiation exposure to patients. Results from mostly asymptomatic, low-risk patients show that the absence of CAC is associated with low probability of significant coronary obstruction (SCO) and a favorable patient outcome.^7^Importantly, however, these findings do not apply to symptomatic, higher-risk populations where SCO may be present in more than 10% of patients with zero calcium score.^8 , 9^

The current investigation was undertaken to identify and validate predictors of SCO in symptomatic patients without coronary artery calcification.

## Methods

### Study design and population

A total of 4,258 individuals from three cohorts were screened for study inclusion. We combined participants without a history of CAD enrolled in the CORE 64 and CORE 320 studies, which have almost identical population characteristics, to determine the prevalence and predictors of SCO in patients without CAC. A single-center registry was then used to validate the model of predictors. Detailed methods are available in supplements.

Briefly, the Coronary Artery Evaluation Using 64-Row Multidetector Computed Tomography Angiography (CORE-64) and the Coronary Artery Evaluation Using 320-row Multidetector Computed Tomography Angiography and Myocardial Perfusion (CORE320) are prospective, multicenter studies that included participants with suspected or known CAD, who were referred for conventional coronary angiography. Detailed description of the CORE-64 and the CORE320 methods have been published elsewhere.^2 , 10^Finally, the Quanta Registry is a real-world registry from patients referred for CTA for clinical purposes in a single center. Participants in the Quanta Registry were included in the validation cohort if they reported any chest pain.

All studies received study approval from their local institutional review boards, and all patients gave written informed consent.

### Acquisition and analysis of coronary artery CT

In CORE64, coronary calcium score imaging was performed using a 64-row CT scanner (Aquilion, Toshiba Medical Systems),^2^and in CORE320, a 320-MDCT scanner (Aquilion ONE, Toshiba Medical Systems).^11^In both cases, the images were analyzed for CAC scoring in a single reading center by experienced readers blinded to the patient clinical information ( Figure 1 ).

**Figure 1:** – Coronary calcium scoring and cardiac computed tomography angiography in a CORE participant with significant coronary obstruction and calcium score zero. A) Coronary calcium scoring. B) Cardiac computed tomography angiography (multiplanar reconstruction). C) Cardiac computed tomography angiography (3D reconstruction).

In the Quanta Registry, all scans were performed using a 128 -row scanner (iCT sp, Philips Healthcare). The scans were reviewed by two experienced readers. A final clinical report was generated for each patient, and each patient was classified as having: a normal scan (no CAD), non-obstructive CAD (<50% stenosis) or obstructive CAD (>50% stenosis) that was also recorded on the database.

### Acquisition and Analysis of Invasive Coronary Angiography Data

In the CORE studies, clinically indicated invasive coronary angiography (ICA) was performed using standard techniques within 60 days of the combined CT acquisition, and lesion severity was determined by quantitative coronary angiography (QCA), as previously described.^2^SCO was defined as an obstruction of 50% or higher as measured by invasive QCA.

### Statistical analysis

Continuous variables were presented as median and interquartile range. Qualitative variables were shown in its frequencies (absolute and relative). The software STATA 10.0 was used for analysis. Data distribution was assessed by graphical methods, including histograms and Q–Q plots.

Initially, univariate and multivariable logistic regression models evaluated cardiovascular (CV) risk factors and anthropometrics as predictors for the absence of CAC (dependent variable) in the entire cohort and in those with SCO. Then, logistic regression univariate and multivariable models evaluated the subset of patients without coronary calcium to assess predictors of SCO (dependent variable). Results of the logistic regression models are shown as Odds Ratio (95% Confidence Interval). Finally, an algorithm was generated for predicting SCO by invasive coronary angiography in patients without coronary calcium by CT and then validated in a different cohort and by 10-fold cross-validation in the original sample.

The total sample size in CORE-64, CORE-320, and Quanta Registry were computed. We included all participants from these studies with available data. Significance level of 5% was used in the analyses.

## Results

### Prevalence and predictors of SCO in patients without coronary calcium

Characteristics of the 509 participants (273 from the CORE64 study and 236 from the CORE320 study) are shown in Table 1 . The majority of enrolled participants were white males in the early seventh decade of life, with a high prevalence of CV risk factors. Of these 509, 117 (23%) had an Agatston calcium score of zero. Two-hundred-fifty-two (49%) of the entire cohort and 13 (11%) of patients without coronary calcium had at least one SCO. Conversely, 392 (64%) of patients with a CAC score ≥ 1 had obstructive coronary artery disease.

**Table 1:** – Patient characteristics in CORE64 and CORE320

Predictor	Median [IQR] or n (%)		

	CORE-64	CORE320	Total

	(n=273)	(n=236)	(n=509)
Age	60 [53. 67]	62 [57. 68]	61 [55. 67]
Male gender	199 (73%)	136 (58%)	335 (66%)
**Ethnicity**			
White	192 (70%)	138 (58%)	330 (65%)
African American	13 (5%)	29 (12%)	42 (8%)
Asian	58 (21%)	66 (28%)	124 (24%)
Other	10 (4%)	3 (1%)	13 (3%)
BMI, Kg/m^2^	27 [25, 30]	27 [24, 31]	27 [25, 30]
Presence of Hypertension	193 (71%)	167 (71%)	360 (71%)
Presence of Diabetes	62 (23%)	88 (37%)	150 (29%)
Presence of Dyslipidemia	165 (60%)	138 (60%)	303 (60%)
**Smoking status**			
Never	119 (44%)	107 (47%)	226 (45%)
Former	108 (40%)	72 (32%)	180 (36%)
Current	46 (17%)	47 (21%)	93 (19%)
Family history of CAD	75 (27%)	96 (42%)	171 (34%)

*BMI: body mass index; CAD: coronary artery disease.*

The subgroup of patients without coronary calcification was generally of a younger age, of female gender, with lower body mass index (BMI), without diabetes, and without dyslipidemia.

Predictors of zero CAC scoring in patients with SCO are shown in Supplementary Table 1 . In this population, younger age and female gender were consistently related to the absence of CAC. CV risk factors did not relate significantly to coronary calcium in patients with SCO.

### Assessing significant coronary obstruction in the absence of coronary calcium

The prediction of SCO by anthropometrics and CV risk factors in patients without coronary calcium is shown in Table 2 . In these findings, SCO was assessed by both CCTA and ICA. Consistently, being a current smoker increased ~3.5 fold the chance for a SCO in patients without coronary calcium. Other CV risk factors were not significantly associated with SCO in this population. Based on these predictive models for CORE64 and CORE320, a clinical algorithm to rule out SCO was created (Supplementary material).

**Table 2:** – Prediction of significant coronary obstruction (≥50%) in participants (CORE 64 and 320 studies combined) with zero calcium score, measured by coronary computed tomography angiography (CTA) and by invasive coronary angiography (ICA)

Predictor	CTA ≥ 50%; n=134		ICA ≥ 50%; n=134	
	
	Odds Ratio (95% CI)		Odds Ratio (95% CI)	
	
	Univariate	Multivariable	Univariate	Multivariadble
Age, years	0.956 (0.900; 1.016)	0.960 (0.888; 1.038)	0.979 (0.927; 1.034)	0.987 (0.924; 1.054)
Male gender	1.922 (0.715; 5.165)	1.382 (0.415; 4.596)	1.977 (0.777; 5.032)	1.558 (0.524; 4.632)
**Ethnicity**				
African American vs. White	0.227 (0.028; 1.839)	0.148 (0.016; 1.342)	0.369 (0.078; 1.741)	0.327 (0.063; 1.684)
Asian vs. White	0.769 (0.230; 2.578)	0.706 (0.162; 3.073)	0.596 (0.182; 1.955)	0.442 (0.108; 1.800)
Other vs. White	3.333 (0.506; 21.970)	4.260 (0.470; 38.601)	0.969 (0.101; 9.276)	1.169 (0.102; 13.336)
BMI, Kg/m^2^	1.019 (0.922; 1.126)	0.986 (0.852; 1.140)	0.991 (0.900; 1.091)	0.939 (0.819; 1.076)
Presence of hypertension	1.727 (0.613; 4.860)	1.977 (0.576; 6.787)	1.361 (0.529; 3.503)	1.602 (0.519; 4.942)
Presence of diabetes	1.831 (0.632; 5.302)	2.329 (0.652; 8.319)	1.917 (0.700; 5.245)	2.458 (0.773; 7.823)
Presence of dyslipidemia	2.132 (0.793; 5.731)	1.932 (0.614; 6.081)	1.150 (0.469; 2.822)	1.160 (0.410; 3.282)
**Smoking status**				
Current vs. Former/Never	3.630 (1.301; 10.128)	3.562 (1.036; 12.246)	3.630 (1.358; 9.706)	3.448 (1.112; 10.691)
Positive family history of CAD	1.677 (0.628; 4.474)	1.862 (0.568; 6.105)	1.293 (0.501; 3.340)	1.347 (0.454; 3.997)
**Overall AUC**		**78 (75-93)**		**73 (70-89)**

*Sample sizes reflect multivariate analysis, where subjects with any covariate missing are excluded. Univariate sample sizes may be somewhat larger. BMI: body mass index; CAD: coronary artery disease.*

In the validation cohort of 742 subjects, only 16 (2.2%) had disease by CTA. The majority (77.2%) had atypical angina, 12.5% had typical angina and the remaining 10.2% had non-angina chest pain, with a generally lower CV risk profile compared to the CORE studies ( Table 3 ). After applying the CORE algorithm, 199 (26.8%) of the 742 subjects were classified as likely having significant coronary disease. Validation results are summarized in Supplementary Table 2 .

**Table 3:** – Characteristics of participants with zero calcium scoring in the CORE studies and validation cohort

Characteristics	Median [IQR] ou n (%)	

	CORE (n=117)	Validation cohort (n=742)
Age, years	56 [51. 62]	52 [43. 61]
Male sex	54 (46%)	246 (33%)
BMI, Kg/m^2^	27 [24. 30]	27 [24. 30]
Hypertension	67 (58%)	322 (43%)
Diabetes	22 (19%)	85 (11%)
Dyslipidemia	54 (46%)	278 (37%)
Current smoker	21 (18%)	59 (8%)
Family history of CAD	34 (29%)	181 (24%)

*BMI: body mass index; CAD: coronary artery disease.*

## Discussion

Our study showed that younger age, female gender, and lower CV risk profile were associated with a zero coronary calcium score in the setting of high-risk patients referred for invasive cardiac catheterization. We also showed that in this population, having a zero calcium score was associated with 83% lower risk of SCO compared to those with any coronary calcification. Importantly, current smoking was the strongest predictor of SCO in patients without coronary calcium.

Coronary disease is a major cause of death worldwide and preventive efforts have been made to diagnose CAD early, as well as to accurately stratify the risk of coronary events.^12^In asymptomatic patients, the presence and grade of coronary calcium has shown to be a strong predictor of CV events;^13^however, the role of coronary calcium for decision-making, particularly in symptomatic patients, is less established.^14^

In accordance with previous reports,^13^we showed that the absence of coronary calcium reduces the probability of SCO. Recent clinical studies, however, suggest that detecting nonobstructive CAD may be at least as important as SCO for guiding patient management.^15 , 16^There remains controversy over the value of coronary calcium scanning to rule out significant coronary disease in symptomatic patients.^17^

Expectedly, we found younger women with chest pain but without known diabetes, dyslipidemia, or hypertension to be more likely to have a zero calcium score. This is a population that is frequently seen in emergency rooms.^17^Grandhi et al.^18^found a prevalence of SCO below 5% in a registry of patients without coronary calcium. However, clinicians may not be able to discharge symptomatic patients based on the absence of coronary calcium alone. It is clear that we need to develop further assessments, such as a clinical score, to better stratify risk in this group of people with zero calcium scoring.^19^

When looking into the parameters associated with SCO in those without coronary calcium, current smoking consistently stands out as a major risk factor. Calcium deposits occur early in the cascade of atherosclerotic disease, but they are detected by imaging methods after later accumulation.^20^Possibly, smoking accelerates coronary disease leading to significant vessel obstruction before calcium deposits. Also, smoking strongly relates to inflammation and thrombosis, which may be important factors related to atherosclerotic plaques’ instability and further sudden vessel obstruction.^21^

Other risk factors, such as age, seems to be relevant when assessing the prognostic ability of calcium score.^22^Therefore, we combined smoking status with age and other risk factors to propose an algorithm that could further stratify risk of SCO in patients with zero calcium score rating. However, the discrimination ability decreased in the validation cohort. The validation lost sensitivity for assessing the relatively rare event of SCO but maintained favorable specificity and negative predictive value. Thus, although the algorithm is not suitable for a wide use at this time, a clinical algorithm may be helpful to better identify patients without coronary calcium that may not undergo a CTA. A clinical trial is desirable to aid establishing the best approach to using calcium scores in a setting of symptomatic patients.

Our study has significant limitations. Despite a relatively large sample, SCO is a rare event in patients without coronary calcium and the low number of outcomes may jeopardize the statistical analysis. Nonetheless, obstructive coronary disease was relatively rare in patients without coronary calcium, and notoriously difficult to predict using clinical variables. There are intrinsic methodological limitations in using the QUANTA Registry as a validation cohort for a risk algorithm developed in the CORE studies. The fact that the algorithm did not perform as well in the validation cohort as it did in the CORE studies might be due to the lower disease prevalence, affecting sensitivity and positive predictive value.

### Ethical approval

All centers involved in CORE-64 and CORE320 received study approval from their local institutional review boards, and all patients gave written informed consent. All patients from the Quanta Registry gave informed consent and authorized the use of their information for research purposes. In addition, the studies were performed in accordance with the ethical standards of the 1964 Declaration of Helsinki and its later amendments or comparable ethical standards.

## Conclusions

The absence of calcium score alone reduces the risk of SCO. Smoking was a major risk factor related to SCO in the absence of coronary calcium. Finally, we proposed a risk score that computes clinical variables, aiming to rule out coronary disease in symptomatic patients with zero calcium scoring. However, further studies are needed to better prove this concept.

## *

Supplemental Materials

For additional information, please click here
